# The Story behind the Science: Preprints of pandemic potential—how bioRxiv and medRxiv brought preprints to the life sciences

**DOI:** 10.1128/mbio.02989-25

**Published:** 2026-01-12

**Authors:** Richard Sever

**Affiliations:** 1 openRxiv Corporation, Davis, California, USA; 2 Cold Spring Harbor Laboratory, New York, New York, USA; Johns Hopkins Bloomberg School of Public Health, Baltimore, Maryland, USA

**Keywords:** preprints, COVID, publishing

## Abstract

The bioRxiv and medRxiv preprint servers brought preprinting to the life sciences and played a critical role in disseminating COVID research during the pandemic. Here, I reflect on the birth of bioRxiv and medRxiv and the crucial role so many members of the community played, our experience during the pandemic, and the launch of the new non-profit organization set up to oversee the servers. The pandemic was a stress test for bioRxiv and medRxiv that demonstrated their value and robustness. Under the umbrella of openRxiv, they are now poised to become long-term infrastructure underpinning a new publishing ecosystem.

## COMMENTARY

When we launched medRxiv in mid-2019, we never could have anticipated that just a few months later, New York would be at the epicenter of a rapidly escalating global pandemic, and we would be handling hundreds of papers about a previously unknown virus every week. Articles about bioRxiv and medRxiv preprints would appear on the *New York Times* front page, and our team worked every waking hour, seven days a week, making rapid decisions about sometimes controversial work and disseminating results of COVID research to millions of readers around the globe. Along the way, we were called dangerous and irresponsible by some, wimps and censors by others, but most in the biomedical community valued the service, and even Anthony Fauci and the head of the WHO called attention to the importance of preprints.

None of this was foreseeable back in 2013, when at home one evening in front of my laptop I hit send on a tweet that announced the launch of bioRxiv to the world. In some ways, that night provided a foretaste of what was to come: there was a storm of supportive tweets amplifying the message, a bunch of queries, and almost immediately a series of attacks. Preprints had been a feature of physics, math, and computational science since arXiv launched in 1991 (1). There had been attempts to kickstart similar behavior among biologists; none had been successful. Opposition from journals had played a part, but many people simply thought that biology was different. They argued physics produced easily verifiable conclusions whereas biology was messy and more competitive, so the peer review filter was essential. Despite this, a few biology preprints were appearing on arXiv and a handful of geneticists were highlighting them on a website called Haldane’s Sieve, created by Graham Coop, a young PI at UC Irvine, and Joe Pickrell, then a postdoc at Harvard. The feeling that the moment for a biology-focused server had arrived began to grow in my mind. “Why now?” was my colleague John Inglis’s response when I brought up the possibility. After all, this was hardly an original idea and one he had first considered as far back as 1998 during a meeting at Cold Spring Harbor Laboratory (CSHL), where we worked. Our colleague Wayne Manos, who had previously worked with the physics community, weighed in, and the more we discussed the idea, the more we began to think the time—and, critically, the place—was right. My view was it was inevitable that the publishing process would move upstream, someone was going to do this, and it should be us.

Discussions with faculty colleagues like Mickey Atwal, Mike Schatz, and Justin Kinney, along with Leonid Kruglyak, a geneticist then at Princeton, all of whom were former physicists, mathematicians, or computational scientists, revealed it was not just Joe and Graham who thought biologists should embrace preprints. They were quick to assure us that physicists were no different from biologists, and there was no reason to think this could not work in biology. Conversations with up-and-coming computational biologists like Daniel MacArthur and Yaniv Erlich confirmed this.

Another person who did not think physicists were outliers was arXiv founder Paul Ginsparg. He snorted at the idea that biology was somehow different and said physicists were just as competitive and the notion that their conclusions were self-evident was nonsense. Another thing he said stuck in our minds: no community that started to post preprints had stopped. Paul would go on to join the bioRxiv Advisory Board, and over the years, we would have many conversations with his colleagues Oya Rieger, Jim Entwood, Stephanie Orphan, Steinn Siggurðson, and Ramin Zabih about ways to align policies and processes. Our first meeting at Cornell in Ithaca was memorable for another reason. A huge storm grounded all flights in the Northeast. So John and I rented a car and drove for six hours through a massive Nor'easter, amid blinding rain and sheet lightning the like of which I have never seen, all the way back to New York City.

Having received arXiv’s blessing, we began to plan the website. We could adapt the components required from a scheme I’d devised for another project, but the question remained: if we built it, would they come? John and I felt strongly that this should be a non-profit, community initiative on the arXiv model. That the initiative arose within CSHL seemed critical. CSHL is a highly respected research institute, but it also has a long history of science communication. Locating bioRxiv within CSHL would give bioRxiv the best chance of success. Leonid, Daniel, Yaniv, Joe, and former *Nature* editor Chris Gunter agreed, and together we hatched a launch plan over lunch at the annual Biology of Genomes meeting. Somehow, Jocelyn Kaiser, a journalist from *Science*, got wind of the gathering, and there was an awkward moment when she tapped on the glass conference room door asking to be let in, and we turned her away—hardly an open access approach!

What would also be needed to get bioRxiv off the ground was backing by CSHL’s President Bruce Stillman, and it was with some trepidation that John presented Bruce with the proposal we had drafted. Somewhat to our surprise, Bruce immediately agreed to the idea. His support was crucial. Not only did he green-light the project, but he became an outspoken advocate for it. It is easy to forget that early on, there were a lot of people who thought bioRxiv was a very bad idea. One even claimed it was the worst thing CSHL had ever done—quite a statement given the place was once a eugenics records office.

A spectrum from support to outright animosity became a pattern that persists to this day. As we worked to build bioRxiv with Stanford spin-off Highwire Press and our colleagues Ted Roeder, Linda Sussman, Inez Sialiano, and Jan Argentine, John and I also began the process of trying to win hearts and minds within the wider scientific and publishing communities. There would be no point in building bioRxiv if no journal would accept a paper that had appeared there, and backroom conversations were needed to win over editors, many of whom vehemently opposed the idea. Conversations with scientists tended to follow a predictable arc: those unfamiliar with preprints were initially skeptical, but when they thought the process through logically, they recognized it made sense; their only real concern was how journals would respond. That was less predictable.

Once we assured publishers that bioRxiv’s mission was simply to decouple dissemination from evaluation and that we were committed to not performing peer review ourselves, many journal editors became enthusiastic. Their support was critical, both in reassuring authors and legitimizing preprints as part of the scientific workflow. Mark Johnston and Tracey DePellegrin at *Genetics*, for example, immediately embraced the idea and became the first partners in the B2J project that automatically sends papers from bioRxiv to journals. Similarly, Olivier Pourquié, James Briscoe, and Katherine Brown at *Development* became big advocates for preprinting and helped create preprint-highlighting services like preLights and In Preprints. Other publishing folk were more cautious. Some were very resistant, and there were spirited public debates with editors from various publishers—Emilie Marcus at *Cell* in particular—which now seem a touch ironic given those same publishers later acquired their own preprint servers. In a few cases, people were outright hostile. I will never forget the time a publishing executive from a clinical society declared during a lunch meeting, “What you’re doing is like giving a loaded gun to a toddler”. The cynic in me continues to believe that arguments from some quarters about the “dangers” of preprints were rooted in commercial rather than societal concerns, though there were good reasons to be cautious when it came to actionable clinical research (see below).

Despite these conflicts, most journals came around. Meanwhile, the scientific community voted with their feet. bioRxiv grew exponentially over the next few years, and new disciplines came on board. Geneticists and evolutionary biologists embraced it immediately. Neuroscience followed swiftly behind. Next came cell and developmental biology. A growing community of enthusiasts and advocates would play a big role. Many of them would join the bioRxiv Affiliate group. Young scientists like Prachee Avasthi, Needhi Bhalla, Elisa Fadda, Michael Hoffman, Stephen Royle, and Nicolai Slavov, frustrated with the existing publishing system, rapidly became activists, and we made a lot of new friends in the expanding biology Twitter network. But bioRxiv was also embraced by later-career scientists. Old friends like Pam Silver, Fiona Watt, Leslie Vosshall, Jonathan Eisen, Alfonso Martinez Arias, and Jim Woodgett were very supportive, and Nobel laureates such as Marty Chalfie and Carol Greider began to tout the benefits, inspired by an ASAPbio meeting in 2017 organized by Ron Vale and Jessica Polka.

Another person paying attention was Cori Bargmann, the recently appointed head of science at the Chan Zuckerberg Initiative (CZI). Central to CZI’s mission was the idea that technology could speed up science. So a website that aimed to do just that sparked her interest. Bumping into our CSHL colleague Terri Grodzicker at a meeting, Cori asked about bioRxiv, and Terri put us in touch. Cori and I met in her office at Rockefeller, and I pitched the idea of a CZI “channel,” a feature we had introduced that aggregated preprints from particular initiatives. Cori was more interested in the big picture, however. She wanted to know what we were doing and how and particularly appreciated the fact that we had put a lot of thought into the screening process. I was struck by how insightful her questions were and the fact that she really got what we were trying to do. I had the same feeling a few weeks later when I met her colleague Jeremy Freeman for lunch in Korea Town. Jeremy personified CZI’s philosophy: a former neuroscientist, he was equally adept at computing and biology. This would be the first of many meetings John, Cori, Jeremy, and I would have and the beginning of a fantastic collaboration. Cori and Jeremy shared many aspects of our vision. Importantly, they also agreed that funding bioRxiv rather than building a new “central server” that simply aggregated content from multiple servers, as another group had proposed, was the way forward. CZI funded bioRxiv, and fortunately, the central server idea was abandoned. I continue to believe it would have been a mistake for the community and would have ushered in a highly fragmented, commercially dominated preprint landscape closely resembling the picture for journals—something almost every scientist we met wanted to avoid.

**Fig 1:**
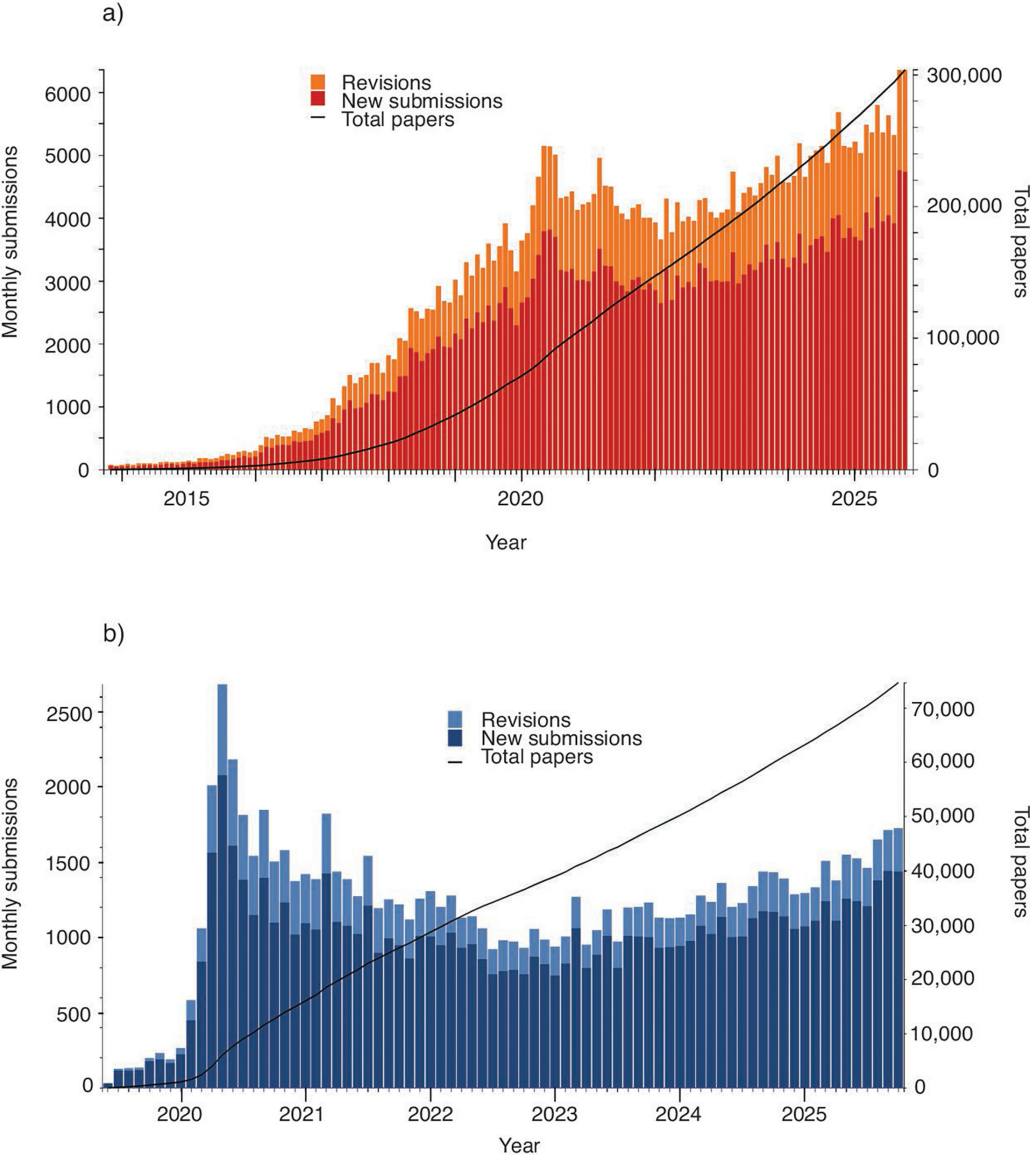
Submissions to bioRxiv (**a**) and medRxiv (**b**) have grown over time. bioRxiv saw a spike early in the pandemic and then a lag that probably reflected shuttering of many labs during this period. medRxiv saw a more significant spike as the server had only just launched and preprinting was a new phenomenon in clinical research; it was dominated by COVID papers for the first 3 years. Colored bars show monthly submissions and relate to the left-hand *y*-axis. Black lines show total papers and correspond to the right-hand *y*-axis.

CZI funding allowed us to grow the service and bring in much-needed additional staff. Sam Hindle joined to lead the content team, and K-J Black came in on the tech side. By 2019, we had posted ~50,000 papers (Fig. 1), and “we just posted this on bioRxiv” was something increasingly heard in conference talks and seminars. Advocacy grew, as did partnerships with groups like PLOS and *eLife*, who saw preprints as the way of the future. John and I wrote a piece with Mike Eisen proposing that funders mandate preprinting (2). Our “Plan U” (for “universal” access) has since been adopted by the Howard Hughes Medical Institute, the Michael J. Fox Foundation, CZI, the Gates Foundation, and Aligning Science Across Parkinson’s, but there is much more to be done on that front. 

Throughout this period, there was one constant, though: no clinical research. That seemed a step too far—or at least too soon. Clinical information was actionable; there were potentially dangerous consequences—who could forget the havoc wrought on public health by the infamous Wakefield paper? (3)—and if we thought convincing biology journals that preprints were a good thing had been difficult, medical journals would be another challenge entirely. Nevertheless, this remained at the back of our minds, and when Eric Topol and Harlan Krumholz penned an op-ed in the *New York Times* in 2015 urging the medical community to embrace preprints, we began to wonder if the time was right (4). We called up Eric and Harlan and agreed on a tentative first step: two new bioRxiv subject categories, Epidemiology and Clinical Trials, with a dedicated screening process in which every paper was looked at by an MD to ensure it was not harmful. Gholson Lyon, a new CSHL faculty member who was a preprint enthusiast, became our first and most active clinical affiliate.

The sky did not fall in; we got plenty of papers, and we began to think clinical preprints could work. “medRxiv” began to crystallize as a concept at CSHL. Meanwhile, Harlan had been convinced of the same thing. Roping in Yale colleague Joe Ross, he began to conceive a “medarXiv,” reaching out to Brian Nosek at the Center for Open Science, who had helped launch preprint servers in various other disciplines. Elsewhere, Theo Bloom and Claire Rawlinson at the *British Medical Journal* (BMJ) had the same idea. BMJ had launched, and then shuttered, a preprint server several years before (5) but enthusiasm for the approach remained. Convinced as we had been before that the community was best served by single, discipline-specific servers, we joined forces. Joe and Harlan brought invaluable clinical expertise, Theo and Claire were experienced medical publishers, and John and I knew how preprints worked. medRxiv would be a CSHL-Yale-BMJ project, complementing bioRxiv but with higher guard rails appropriate for clinical content.

Inevitably, when three organizations are involved, things take longer than you hope. But after all the lawyers were satisfied, medRxiv finally launched in mid-2019. We built the website with Highwire as before, and experienced editors Emma Ganley and John Fletcher came on board to help Sam implement those higher guard rails: additional scrutiny by healthcare professionals such as Gholson, Richard Lehman, and Javier Mancilla Galindo; mandatory ethics declarations; clinical trial IDs; and firm criteria for what we would post as a preprint and what we would decline as “better after peer review.” BAPR became a heavily used abbreviation, one we’d count on many times the following year.

Then came 2020. Reports started to emerge of an outbreak in China, and the first preprint on what was then called “Wuhan novel coronavirus” (6) arrived on 19 January. In the weeks that followed, we would receive reports on how the virus entered cells, models of transmission, and clinical characteristics of the disease. It still seemed a long way away, but at a meeting in Canada in early February, I first heard someone cough and chuckle, “Don’t worry; it’s not coronavirus.” I began to think it would not be long before that was not a joke.

As the world watched events in China, more and more papers on COVID arrived, and everyone began to realize this was not just another outbreak a long way away that would be contained. On 3 March, I took what would be my last flight for 2 years to a meeting with CZI in the Bay Area. John and I met with K-J and new team member Sol Fereres Rappoport in San Francisco that evening, and the next day, together with our Finance Director Steve Nussbaum, we headed down to Redwood City to CZI’s headquarters to meet Dario Taraborelli (Jeremy’s replacement at CZI) and his colleagues. Halfway through the meeting, we had to evacuate the building as it emerged that two people had just tested positive for the new virus (back then, of course, little was known about how it was transmitted). We dined that evening with Alison Mudditt and Veronique Kiermer from PLOS at a restaurant in San Francisco while people around us joked about elbow bumps. I boarded my return flight from SFO the next morning as people around me nervously sterilized their seats with alcohol wipes (no masks back then). I arrived back at Kennedy airport in the evening, drove home, and did not go back to my office at CSHL until over a year later, by which time the scientific community had developed an effective vaccine and bioRxiv/medRxiv had posted more than 20,000 COVID papers.

Our team quickly adapted to remote working as the world locked down. New York was a scary place to be. Case numbers exploded, emergency hospitals were hastily constructed, and trucks full of corpses lined streets because the morgues were full. Despite taking every precaution possible, naturally we worried that we too might be struck down. I vividly remember Theo saying on our weekly medRxiv call, “What happens when we all get sick?” We had to consider that. Meanwhile, medRxiv submissions served as a global tracker for the pandemic. We received a wave from China, then a wave from Italy, and then the United States. Soon there was barely a corner of the globe that had not sent a paper on COVID to bioRxiv or medRxiv. One thing we were all humbled by was the way in which medRxiv served as a place where the local impact of COVID could be reported, be it by a public health agency in a remote region of Africa or scientists nearer home in New York City.

I settled into a daily routine of waking up, switching on my computer, checking Twitter for the latest pandemic news, and then logging in papers all day. Sam, Sol, Theo, and I became paper-processing factories, together with Jan, Inez, and colleagues Barbara Acosta, Joanne McFadden, and Dorothy Oddo, who had been roped in from other projects to handle the deluge of emails from authors. Ted, Linda, and K-J worked continually to keep the tech going. Meanwhile, there was constant back and forth with John, who bore the additional burden of having to run an entire CSHL division remotely. Day merged with day; weekends became indistinguishable from weekdays; the only constant was a steady flow of papers. Just when I began to worry we would all burn out, CZI again stepped up with funding, enabling us to recruit Sanchari Ghosh, Dinar Yunusov, Marisol Muñoz and, later, Olaya Fernández Gayol to help share the load. Meanwhile, former *Cell* editor Angela Andersen connected us with a group of former journal editors who volunteered to help out as freelancers.

Zoom kept us in constant contact with colleagues at home and abroad, and like everyone else, we began to use it to keep up with friends and family. I couldn’t help but note the contrast between bored friends at home trying to find things to do in lockdown and the bioRxiv/medRxiv team, who were working every waking hour, but talking with a physician friend who spent all his days intubating COVID patients put things into perspective. As Theo said on one of our calls, “At least we’re contributing something.” A weekly Zoom call with a group of friends over in England was one thing that kept me going. Most of it was spent chatting about soccer or politics, but it often started off with urgent questions for me about COVID. The pandemic gave unprecedented public attention to science and to preprints in particular. Like many scientists, I found myself explaining immunology to people who had never thought about it before. It was particularly bizarre when the questions were about news reports based on a preprint that appeared on bioRxiv or medRxiv that week.

The papers kept coming. We received everything from studies of the structure of the Spike protein (7) to the immune response, emergence of different variants and the waves of infection they drove across the globe, and ultimately reports of therapies. A triumph was the paper from Martin Landray’s team in the United Kingdom on the successful dexamethasone trial for COVID (8). Early availability of the preprint meant that undoubtedly lives were saved. Mike Joyner and Arturo Casadevall told me the same about preprints on convalescent plasma (9). For years, we had been citing a paper by *PLoS* editor Larry Peiperl touting the potential of preprints for rapid sharing of information during disease outbreaks (10). That now seemed remarkably prescient.

The pandemic writ large the debate around biomedical preprints. We were simultaneously accused of gross irresponsibility for posting any COVID preprints at all and for not posting every one submitted. Some argued dissemination of un-peer-reviewed material was just too dangerous; others argued that declining anything was censorship. John tended to receive the indignant emails; I got the Twitter “reply guys.” We were both attacked by people claiming that medical misinformation on medRxiv would kill people. Meanwhile, an eminent biologist called us “wimps” for turning away predictions of COVID treatments that might have dangerous unintended consequences, and one British MD told us we had blood on our hands for not posting his endorsement of hydroxychloroquine.

Aggrieved and eccentric authors are a problem familiar to any editor. We have always gotten a fair bit of pseudoscience, and there is a lot of stuff sent in that is plain nuts (a proposed geological rather than virological origin of COVID was perhaps my favorite). Both bioRxiv and medRxiv have always had a “do no harm” policy under which we decline papers that might jeopardize public health measures, but concerns about misinformation and the potential impact of flawed work became more acute with COVID (11). This was something journals and preprint servers both faced, and while some detractors pointed the finger at preprints, much of the time the problematic work appeared in journals (12, 13). bioRxiv and medRxiv at least had a “not peer-reviewed” disclaimer on every paper (something journalists increasingly were noting) and the option of declaring some submissions BAPR. One thing we worried about was runs on over-the-counter meds and drugs widely prescribed for other conditions. We had also begun to receive various suspect computational papers predicting alternative medicines to be effective COVID treatments. In the end, we took what seemed the fairest decision: to exclude all purely *in silico* predictions for COVID therapy. This was generally applauded by scientists and clinicians, who noted that serious researchers would provide supporting *in vitro* work.

To limit subjective decision-making and maximize signal-to-noise, we had excluded opinion pieces at bioRxiv and medRxiv from the outset. COVID underscored the wisdom of that decision. We received all sorts of hypotheses about COVID epidemiology and treatment, and it was simply much easier to say “research articles only” than argue with every wacko or conspiracy theorist. I had always been a little uncomfortable with our “no theories/reviews” policy, despite being its loudest proponent. Indeed, John and I were both unsure about this initially, but it seemed the right call. By the time the COVID pieces started rolling in, we were certain it was. It did not stop the attacks though, some of which occasionally reached fever pitch when it was claimed we were censoring papers on COVID operations, outcomes, or origins, or submissions from certain people. Perhaps the most ludicrous accusation was that bioRxiv rejected review articles if they were written by women!

Another potential issue was dual-use research of concern (DURC)—for example, research creating so-called pathogens of pandemic potential (PPPs). I had spoken at the National Science Advisory Board for Biosecurity about this a few years earlier and explained that such papers would fall into our BAPR category. But what constitutes a PPP if the pathogen is already causing a pandemic? Valda Vinson at *Science* was the first to raise this question. Given that many labs had pivoted to SARS-CoV-2 research to aid the pandemic effort, there was also the possibility that one might cross the line accidentally or out of ignorance. We coordinated a call to discuss the issue with Valda and her colleagues, together with experts like Arturo and Marc Lipsitch, who had considered this issue for the American Society for Microbiology. Marc, Arturo, and later Mike Imperiale agreed to advise on any papers we were unsure about. In the end, there were few issues. And with the emergence of new SARS-CoV-2 variants, it was pretty clear the natural experiment of running the virus through a few billion people was enhancing the virus more than any antibody-challenge papers we might receive.

The arrival of the vaccines was naturally an occasion for celebration but also presented challenges. Like so many New Yorkers, I spent hours crouched over a laptop hitting refresh constantly to get an appointment. Eventually, a spot in Harlem came up. I got my booster a few weeks later in the Bronx and even did a Facebook Live event for a New York state senator to explain the benefits of vaccination to the public. Meanwhile, we began to get papers on the vaccines at medRxiv. Inevitably, there were some dubious submissions from anti-vaxxers. Fortunately, this was a scenario we had foreseen and a clear case of applying our rule of not posting papers that could jeopardize an accepted public health measure. At the same time, the comment section was deluged. Commenting on papers is something we introduced from the get-go, but as many have noted over the years, commenting on papers is relatively uncommon and scientific culture has not yet adapted to this form of feedback. That was not true of vaccine papers. We got dozens of comments on some papers, and the decision to adopt pre-moderation and firm posting criteria (nothing offensive, nothing irrelevant, nothing ad hominem) was clearly the right one.

**Fig 2:**
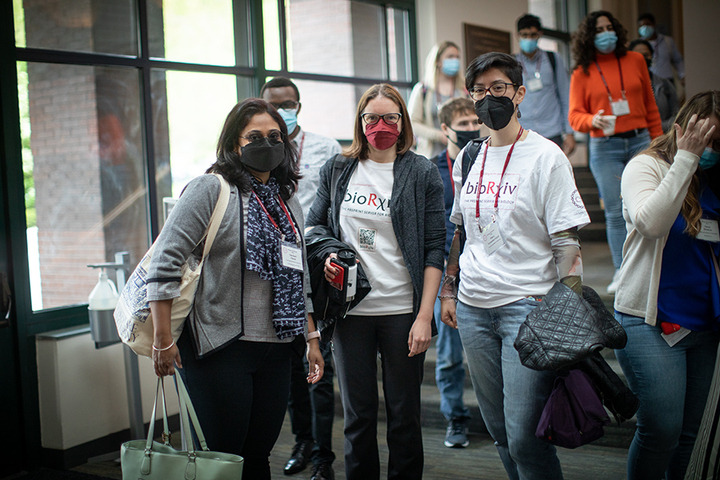
bioRxiv/medRxiv team members Sanchari Ghosh, Samantha Hindle, and Olaya Fernández Gayol attend one of the first in-person scientific meetings to resume at Cold Spring Harbor Laboratory after the pandemic.

The vaccines, of course, made us more comfortable with interacting in person again (Fig. 2). We all returned to the office, and two and a half years after that trip in spring of 2020, John and I again took a flight to California for a CZI meeting. It was wonderful to see bioRxiv supporters like Dario, Jessica, Jaime Fraser, and Casey Greene in real life after so long. A particularly emotional moment was when we greeted our medRxiv colleague Harlan. It was the first time we had seen him in person in three years and more than 150 Zoom calls. That scene would be repeated with Joe, Theo, and Claire at subsequent meetings. Seeing them all in person after so long was very moving, and the gap was a reminder of what everyone had been through.

As the pandemic receded, we’d see more old friends again in real life. Debates with Mike, Casey, and Prachee about the future of publishing now took place at A’s games, not over Twitter, and we finally got to meet all the new bioRxiv and medRxiv supporters we had hitherto only talked with on Zoom. As 2023 passed into 2024, the preprints kept coming, plus there were more of them. Josh Sinanan and later Martina Proietti Onori and Emma Croushore would join the team to help cope with the increase in submissions, and for the first time since 2020, non-COVID submissions on medRxiv overtook COVID submissions (COVID submissions were always only a small fraction of the papers on bioRxiv). While the pandemic became an increasingly distant memory, it was still very clear that it had been both a critical stress test for bioRxiv and medRxiv and a real demonstration of the value of preprints. Many scientists said they found it hard to imagine how SARS-CoV-2 research could have moved so fast without preprints, and more and more began to be convinced this should be the norm for all research. Harlan put it nicely, “We must show preprints are as important for cardiology and cancer as they were for COVID.” The growth in non-COVID papers on medRxiv resembles the first few years of bioRxiv, which makes me optimistic we can (Fig. 1b).

The idea that bioRxiv/medRxiv had survived a critical stress test, that the preprint “experiment” had succeeded, raised the question of what our operation should ultimately look like. Preprints had always been a side project to which we all devoted some of our time. For many of us that “some” had become “most,” and John and I had always thought that eventually, the project would need to grow up and leave the nest. Steve Quake, Cori’s successor at CZI, felt the same way. He and his colleagues Dario and Carly Strasser suggested that the best way forward would be to set up a new non-profit to oversee the servers. Bruce agreed, and the serious discussions about what that organization should look like began. Again, lawyers were involved, again the process took time, but by 2024 we had a plan: a 501(c)(3) non-profit corporation called openRxiv. I would join openRxiv along with colleagues who worked on the servers; John would step back from operations but chair the Scientific & Medical Advisory Board (SMAB). We would hire an experienced CEO and appoint a board of directors including key figures from the scientific community. This would maintain continuity but also ensure that the wider community was represented and that we brought in operational expertise critical for a new organization.

openRxiv finally launched in 2025. The board included Scott Fraser from CZI, Bruce and Harlan; Theo and Joe joined John on the SMAB, together with a fantastic group of biomedical scientists from around the world, some of whom, like Needhi, Daniel, Leslie, Javier, and Fiona, had been with us since almost the beginning. Tracy Teal, a highly experienced open science leader, came in as CEO. Not only was she highly qualified, but she had worked with our team during the transition from CSHL to openRxiv and immediately gelled with everyone. Tracy will help us build the organization and create a stable long-term financial model. One of the arguments for preprints is that they cost significantly less than journal articles ($10^1^/paper vs $10^3^/paper), but those costs add up at high volume. We are fortunate to have support from philanthropic organizations and a growing number of universities and research institutions. Expanding support among the many stakeholders and using technology effectively to increase efficiency will put openRxiv on firm footing so we can play a part in shaping the future of research communication.

What will that future look like? More preprints, and therefore faster science. Steve Quake estimated that if everyone were to post preprints, then we could speed up science fivefold (14). So that’s the main goal. Only ~12% of biologists currently post preprints. There are clearly more hearts and minds to win, but as Paul Ginsparg said, when a discipline adopts preprints, they do not look back. Preprints also represent an opportunity to rethink how we share and evaluate research (15). They do not have the same constraints as journal articles, so what constitutes an article may evolve. The fact that preprints can be revised challenges the traditional notion of a static “version of record,” and maybe we can do a better job of integrating articles with other outputs—data, code, protocols, and so forth. Preprints are also stimulating experiments in peer review—from portable review to community review to AI-based review—so they could actually improve how we assess papers. Meanwhile, funders and institutions are using the opportunity to explore better ways of evaluating people (16), noting that preprints could help rid us of some of the dubious quality proxies used in academia. openRxiv will be at the heart of these experiments. We want to be an enabler that helps improve science communication. In the post-pandemic period, preprints have the potential not only to make science faster but to make it better.
